# RVM+: An AI-Driven Vision Sensor Framework for High-Precision, Real-Time Video Portrait Segmentation with Enhanced Temporal Consistency and Optimized Model Design

**DOI:** 10.3390/s25051278

**Published:** 2025-02-20

**Authors:** Na Tang, Yuehui Liao, Yu Chen, Guang Yang, Xiaobo Lai, Jing Chen

**Affiliations:** 1 School of Medical Technology and Information Engineering, Zhejiang Chinese Medical University, Hangzhou 310053, China; 2 Bioengineering Department and Imperial-X, Imperial College London, London W12 7SL, UK; 3 National Heart and Lung Institute, Imperial College London, London SW7 2AZ, UK; 4 Cardiovascular Research Centre, Royal Brompton Hospital, London SW3 6NP, UK; 5 School of Biomedical Engineering & Imaging Sciences, King’s College London, London WC2R 2LS, UK

**Keywords:** video portrait segmentation, temporal consistency, knowledge distillation, AI-enabled vision sensors, real-time processing, dynamic environments

## Abstract

Video portrait segmentation is essential for intelligent sensing systems, including human-computer interaction, autonomous navigation, and augmented reality. However, dynamic video environments introduce significant challenges, such as temporal variations, occlusions, and computational constraints. This study introduces RVM+, an enhanced video segmentation framework based on the Robust Video Matting (RVM) architecture. By incorporating Convolutional Gated Recurrent Units (ConvGRU), RVM+ improves temporal consistency and captures intricate temporal dynamics across video frames. Additionally, a novel knowledge distillation strategy reduces computational demands while maintaining high segmentation accuracy, making the framework ideal for real-time applications in resource-constrained environments. Comprehensive evaluations on challenging datasets show that RVM+ outperforms state-of-the-art methods in both segmentation accuracy and temporal consistency. Key performance indicators such as MIoU, SAD, and dtSSD effectively verify the robustness and efficiency of the model. The integration of knowledge distillation ensures a streamlined and effective design with negligible accuracy trade-offs, highlighting its suitability for practical deployment. This study makes significant strides in intelligent sensor technology, providing a high-performance, efficient, and scalable solution for video segmentation. RVM+ offers potential for applications in fields such as augmented reality, robotics, and real-time video analysis, while also advancing the development of AI-enabled vision sensors.

## 1. Introduction

Video portrait segmentation is the process of accurately distinguishing between foreground and background regions in video frames [1]. It plays a crucial role in diverse applications, including human-computer interaction, augmented reality (AR), video editing, autonomous navigation, and video surveillance. The exponential growth in video content has increased the demand for advanced segmentation technologies that can deliver both high accuracy and computational efficiency in dynamic scenarios [2,3]. However, achieving reliable segmentation in video sequences is challenging due to factors such as temporal variations, occlusions, deformations, and the need for real-time processing in resource-constrained environments.

Pixel-level image segmentation (ISS) forms the foundation for video segmentation by analyzing features such as contrast, texture, and grayscale values for pixel-wise classification [4,5]. ISS is crucial for various applications, including pedestrian detection, intelligent rescue operations, medical imaging, and autonomous driving [6,7]. While significant progress has been made in static image segmentation, extending these techniques to video introduces additional complexities. Ensuring temporal consistency across frames and handling rapid motion, occlusions, and dynamic backgrounds require more sophisticated algorithms [8,9].

Deep learning has revolutionized semantic segmentation over the past decade. Fully Convolutional Networks (FCNs) replaced fully connected layers with convolutional layers [10], enabling pixel-level classification. Architectures such as U-Net [11] and SegNet [12] introduced hierarchical feature extraction and upsampling, enhancing resolution and performance. Further innovations, such as pyramid pooling in PSPNet [13] and atrous convolutions in DeepLab [8], have improved segmentation by integrating multiscale features and global context.

Lightweight architectures have become increasingly popular for balancing accuracy and computational efficiency. ENet optimized depth while maintaining speed [14], ICNet utilized multi-resolution branches [15], and BiSeNet combined detail and semantic branches for real-time segmentation [16]. MobileNet-v2 leveraged depthwise separable convolutions for lightweight, efficient models [17]. While these architectures perform well in resource-constrained scenarios, they often struggle to maintain temporal consistency and robustness in dynamic video environments.

The challenges of video segmentation are further amplified in scenarios with significant motion, multi-subject overlaps, or complex backgrounds [18,19]. Many existing models fail to maintain frame-to-frame consistency, particularly in long sequences with intricate temporal dynamics. Additionally, their high computational demands hinder real-time applicability.

The Robust High-Resolution Video Matting (RVM) framework introduced temporal guidance to improve segmentation in dynamic video data [3,20]. However, this method still struggles with vanishing gradients in long sequences and performance drops in overlapping or fast-moving scenarios.

To address these limitations, this study introduces RVM+, an enhanced framework built upon the RVM architecture. The key innovation is the integration of Convolutional Gated Recurrent Units (ConvGRU) to improve temporal modeling. ConvGRUs effectively capture dependencies between frames, retaining both spatial and temporal features, ensuring temporal consistency, and achieving accurate segmentation even in dynamic scenarios. Additionally, we propose a knowledge distillation strategy to optimize the RVM+ model. By transferring knowledge from a fully trained teacher model to a smaller student model, computational requirements are reduced without compromising accuracy. This optimization is crucial for real-time applications on resource-constrained platforms such as mobile devices and edge systems. Rigorous evaluations on multiple challenging datasets confirm that RVM+ outperforms state-of-the-art methods. Finally, we use key metrics such as Mean Intersection over Union (MIoU), Sum of Absolute Differences (SAD), and temporal-specific measures (dtSSD) to validate the model’s efficiency and temporal consistency.

This work makes the following contributions:

(1) Development of the RVM+ model: By integrating ConvGRU into the RVM framework, this study represents a significant advancement in video segmentation technology, particularly in handling dynamic content and temporal changes.
(2) Optimization through knowledge distillation: The study introduces an innovative approach to model optimization using knowledge distillation, effectively reducing model size and computational requirements—critical for real-time processing applications.
(3) Comprehensive performance evaluation: Extensive testing on a variety of challenging datasets demonstrates the enhanced segmentation capabilities of the RVM+ model, setting new benchmarks for accuracy compared to existing segmentation models.

The remainder of this paper is organized as follows: Section 2 provides a detailed explanation of the RVM+ framework and its methodology. Section 3 presents experimental results, followed by a discussion of findings in Section 4. Finally, Section 5 concludes the paper by summarizing the contributions and outlining future research directions.

## 2. Materials and Methods

**Figure 1:**
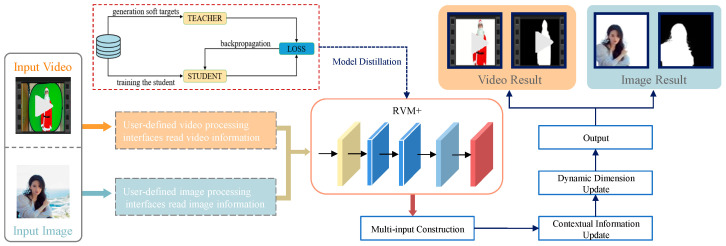
Flowchart of our video portrait segmentation structure.

This study outlines a systematic approach to developing an advanced video portrait segmentation algorithm. The process begins with the collection and augmentation of diverse datasets containing portrait images and video sequences, forming a robust foundation for training and testing. A balanced subset of this data, incorporating both static and dynamic content, is then selected for effective algorithm training. A key innovation in this workflow is the use of knowledge distillation to optimize the model’s size and speed, making it suitable for real-time applications. The process concludes with a comprehensive evaluation of the model, benchmarking its accuracy, efficiency, and robustness against existing methods. This entire workflow, depicted in Figure 1, is designed to yield a highly efficient and accurate model for video portrait segmentation.

### 2.1. Datasets and Preprocessing

In this study, we developed the VM-datasets for video portrait segmentation, focusing on capturing a wide range of real-world scenarios. Our approach to assembling these datasets was multifaceted and methodically structured to ensure both diversity and realism.

Organization of Open-Source Datasets: The first step was to aggregate data from various open-source web datasets, selecting images that represented a broad spectrum of demographics, backgrounds, and scenarios. Acknowledging the limitations of these datasets, particularly in multi-subject scenes and complex backgrounds, we strategically augmented our collection with an additional 100 images, referred to as MulData [21,22,23,24,25,26,27]. These images were specifically chosen to fill gaps and ensure comprehensive representation of various portrait settings. Furthermore, to enhance the dataset for video applications, we added 2000 unique non-human background images to provide a richer variety of environmental contexts.

**Figure 2:**
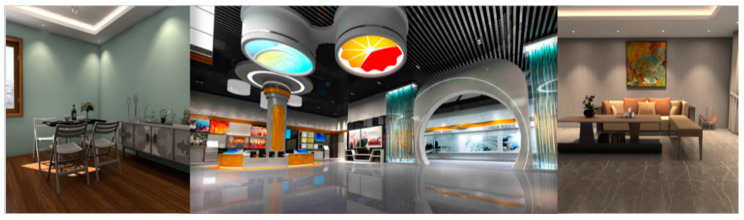
Static background sample images.

**Figure 3:**
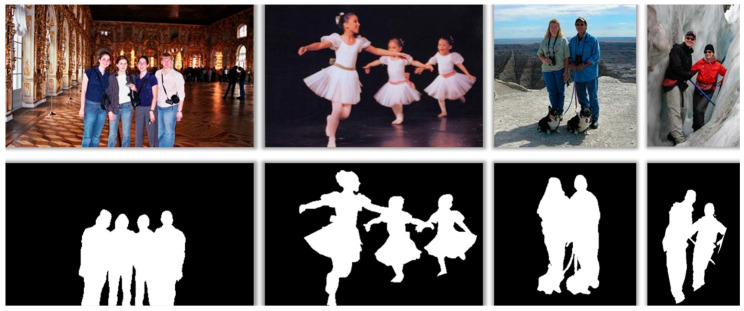
MulData dataset sample images.

This careful curation process is illustrated in Figure 2 and Figure 3, which showcase the diversity of the added data. We also filtered out irrelevant data during the collection process to maintain the dataset’s quality and relevance. Table 1 provides the names and descriptions of the datasets used for training and testing the proposed algorithm.

**Figure 4:**
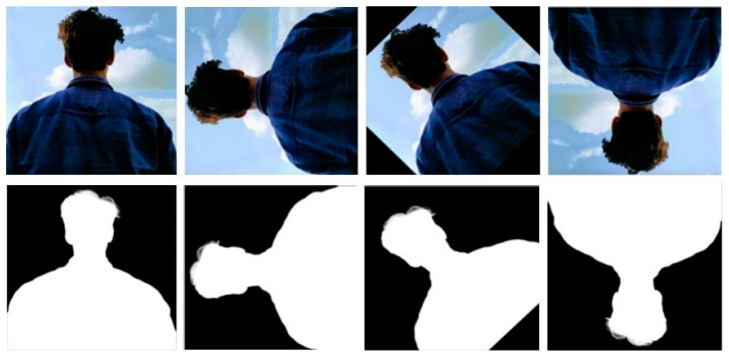
Results of random rotation.

**Figure 5:**
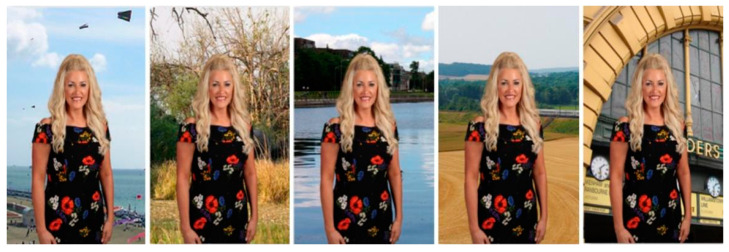
Results of background fitting.

Data Enhancements: To further enrich our dataset, we employed a series of data enhancement techniques designed to mimic real-world variability. These enhancements included motion-related changes, such as scaling and rotation (Figure 4), as well as color adjustments (e.g., brightness and saturation), applied to preserve the natural integrity of the images [28]. To introduce temporal diversity in the video data, we incorporated techniques such as clip reversal and speed variations. Additionally, for static datasets such as VMD and PhotoMatte85, we created composite images by combining transparent background subject images with various backgrounds from the BG dataset (Figure 5). Each subject image was paired with 20 different backgrounds to ensure a realistic and representative mix. This process expanded PhotoMatte85 to 13,165 images and VMD to 89,227 images, simulating a wide range of portrait environments. Each step of our dataset assembly and augmentation was systematically structured and executed with the goal of developing a robust, diverse, and realistic dataset—essential for effective training and testing of our video portrait segmentation model.

### 2.2. Design of the Model Network Structure

Our study focuses on developing a video portrait segmentation model, with an emphasis on the evolution of video portrait processing algorithms. We provide an in-depth analysis of the RVM model’s structure and detail the specific enhancements and adjustments made to optimize its performance.

#### 2.2.1. General Framework of the RVM

**Figure 6:**
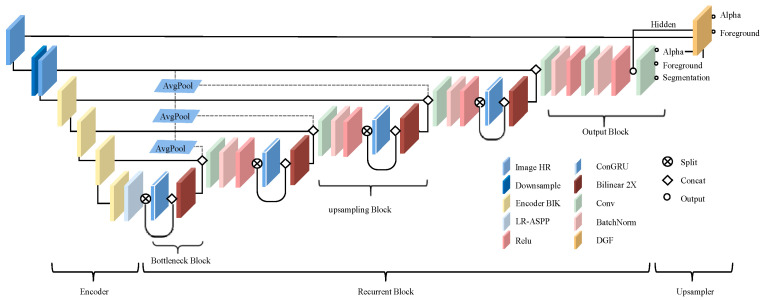
RVM model structure.

The RVM model functions as a real-time foreground segmentation framework for video scenes, employing a U-shaped architecture. It incorporates MobileNetV3-Large as an efficient backbone and utilizes the LR-ASPP module of MobileNetV3 for semantic segmentation tasks. The model architecture consists of a three-part encoder for frame feature extraction, a cyclic decoder for temporal information aggregation, and a depth-guided filter module for high-resolution upsampling, as illustrated in Figure 6.

When processing high-resolution video, the model first downsamples the input to manage resolution and computational load effectively. The encoder then extracts frame features at multiple scales, generating a detailed feature map. These features are passed to the recurrent decoder, where temporal information plays a crucial role.

#### 2.2.2. Structure of the ConvGRU

**Figure 7:**
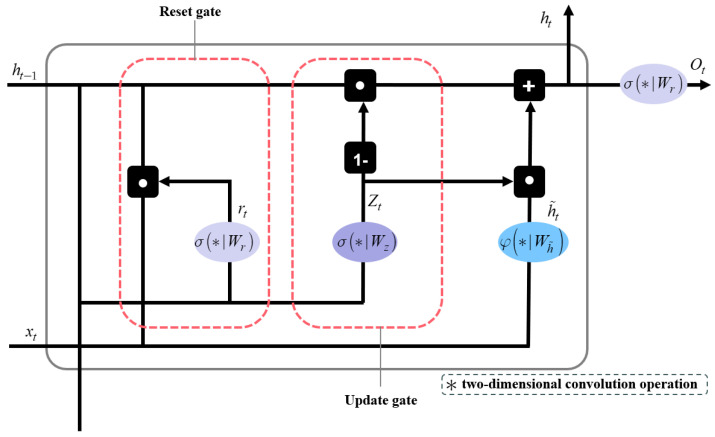
Structure of the ConvGRU module for extracting temporal features.

The ConvGRU, an integral component of the RVM+ model, represents an advanced evolution in video segmentation, particularly effective for tasks requiring temporal sequencing [29]. The design of ConvGRU is characterized by its dual ability to retain relevant information from previous inputs while simultaneously processing new data. This functionality is crucial for accurately contextualizing each frame in a video sequence. ConvGRU selectively retains important information for future frames while discarding data from distant past frames that are no longer relevant. This selective retention is key to the module’s efficiency, as illustrated in Figure 7. By incorporating convolutional layers into its architecture, ConvGRU effectively handles both spatial and temporal data, which is highly advantageous for real-time applications requiring a nuanced understanding of complex video content. The ConvGRU is calculated as follows:

(1) $$rt=\sigma (Wr*[h_{t-1},xt]),$$

(2) $$zt=\sigma (Wz*[h_{t-1},xt]),$$

(3) $$\tilde{h}t=\tanh(W\tilde{h}*[rt⊙h_{t-1},xt]),$$

(4) $$ht=(1-zt)⊙h_{t-1}+zt⊙\tilde{h}t,$$

(5) ot=σ(Wo∗ht).

where xt represents the input data at the current time step, and ht represents the network’s memory or state at the current time step, which depends on the previous hidden state $h_{t-1}$ and the current input xt. σ denotes the sigmoid function, ∗ is the standard two-dimensional convolution operation, and ⊙ denotes the Hadamard product. The parameters Wr, Wz, $W\tilde{h}$, and Wo are the corresponding 3 × 3 convolution kernels.

#### 2.2.3. Structure of the Proposed RVM+

**Figure 8:**
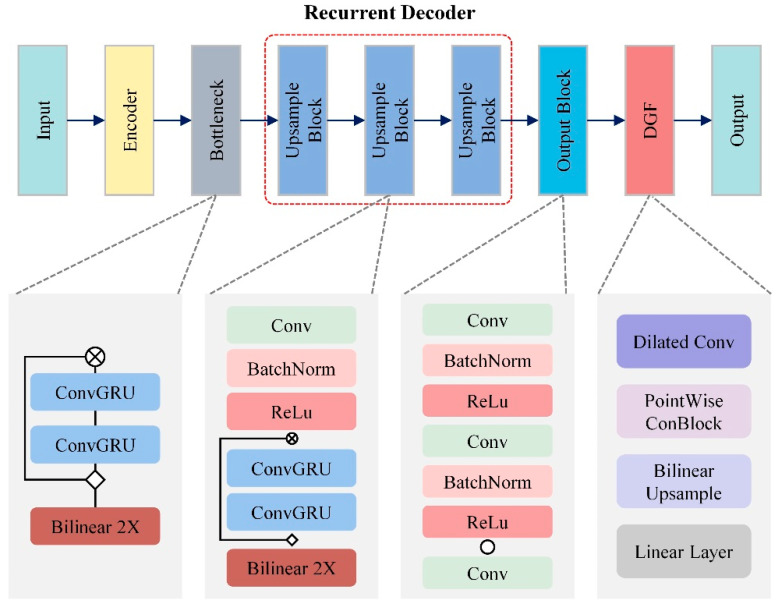
Structure of the RVM+ model.

In the RVM+ model, an advanced iteration of the RVM framework, ConvGRU is strategically integrated to address the specific challenges of portrait segmentation in video. At the bottleneck stage of the model, ConvGRU is crucial for retaining and accurately processing essential temporal information within this compressed segment. This stage plays a key role in distilling significant features from the input data while preserving their temporal coherence. During upsampling, as spatial resolution is progressively restored, ConvGRU modules are vital for reintroducing and refining temporal details. This is essential for maintaining the integrity of time-sensitive features in dynamic scenes, as illustrated in Figure 8.

The effectiveness of the RVM+ model is significantly enhanced by the role of ConvGRU in maintaining temporal continuity, a crucial factor for understanding each frame in its proper context. This is especially important in videos with constantly changing subjects and backgrounds. The model’s feature propagation mechanism enables accurate segmentation across multiple frames, even in complex scenarios involving overlapping objects, variable lighting, and other challenging conditions. This capability is invaluable for high-precision applications such as advanced video editing, augmented reality, and real-time surveillance systems.

Moreover, the RVM+ model emphasizes computational efficiency, which is essential for applications requiring real-time processing, such as live video streaming or interactive AR/VR environments. The efficient ConvGRU modules within the RVM+ model’s streamlined architecture ensure rapid processing of video frames without compromising accuracy, making the model particularly well-suited for environments with limited computing resources.

#### 2.2.4. Loss Function

The model utilizes the Laplace pyramid loss for the alpha map of each frame in the video, with an additional temporal correlation loss introduced to account for the temporal nature of the video. The total loss is defined as follows:

(6) $$L_{l1}^{\alpha }={\left\|\alpha_{t}-\alpha_{t}^{*}\right\|}_{1}$$

(7) $$L_{lap}^{\alpha }=\sum_{s=1}^{5}\frac{2^{s-1}}{5}{\left\|L_{pyr}^{s}(\alpha_{t})-L_{pyr}^{s}(\alpha_{t}^{*})\right\|}_{1}$$

(8) $$L_{tc}^{\alpha }={\left\|\frac{d\alpha_{t}}{dt}-\frac{d\alpha_{t}^{*}}{dt}\right\|}_{2}$$

where $\alpha_{t}$ is the test map and $\alpha_{t}^{*}$ is the true label.

To learn foreground $F_{t}$ and true label $F_{t}^{*}$, *L*_1_ loss $L_{l1}^{F}$ and temporal correlation loss $L_{tc}^{F}$ are proposed, where $\alpha_{t}^{*}>0$, whose loss is defined as follows:

(9) $$L_{l1}^{F}={\left\|\left(\alpha_{t}^{*}>0)*(F_{t}-F_{t}^{*}\right)\right\|}_{1}$$

(10) $$L_{tc}^{F}={\left\|\left(\alpha_{t}^{*}>0)*(\frac{dF_{t}}{dt}-\frac{dF_{t}^{*}}{dt}\right)\right\|}_{2}$$

Thus, the overall loss function can be described as follows:

(11) $$L^{M}=L_{l1}^{\alpha }+L_{lap}^{\alpha }+5L_{tc}^{\alpha }+L_{l1}^{F}+5L_{tc}^{F}$$

For semantic segmentation, the network is trained solely on the human category. To learn the segmentation probability $S_{t}$ and the true binary label $S_{t}^{*}$, we compute the binary cross-entropy loss as follows:

(12) $$L^{S}=S_{t}^{*}(-\log(S_{t}))+(1-S_{t}^{*})(-\log(1-S_{t}))$$

### 2.3. Model Knowledge Distillation Strategy

**Figure 9:**
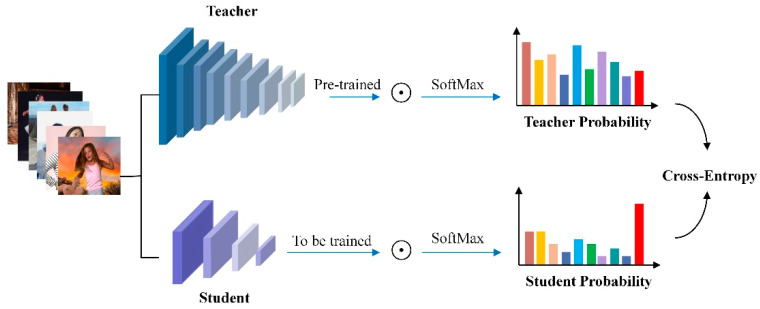
Specific schematic of knowledge distillation.

Our study employs a knowledge distillation process to create a compact yet robust model, particularly suited for complex image segmentation tasks [30]. As illustrated in Figure 9, this process begins by using the RVM+ model as a “teacher”, which, once fully trained, captures intricate relationships within the dataset. The predictions of the teacher model, referred to as “soft targets”, are essential for transferring knowledge to a smaller, simpler “student” model. To enhance the effectiveness of this transfer, we incorporate a label transfer data mining method and introduce a temperature variable *T*. This approach accurately represents label relationships, facilitating more efficient knowledge transfer. The training of the student model involves a combination of insights from the teacher model and a specialized distillation loss function that balances the KLDiv loss for soft targets and the BCE loss for real labels. During online prediction, the student model operates at a reduced temperature setting (*T* = 5), optimizing the extraction of distilled knowledge from the teacher model.

This distillation technique is an integral part of our training methodology, enabling the development of an efficient yet powerful model. It compresses the rich knowledge of the teacher model into the smaller student model, effectively overcoming the challenges associated with large-scale models while leveraging their strengths in a resource-optimized manner. The temperature variable *T* plays a crucial role in influencing the probability distribution during this process. Together, these elements ensure a comprehensive and effective approach to training the student model, addressing the limitations of large-scale models while exploiting their advantages in a more resource-efficient manner.

## 3. Results

### 3.1. Evaluation Metrics

To evaluate the accuracy of image and video segmentation networks in our study, we use a comprehensive set of metrics, categorized into pixel accuracy and Intersection over Union (IoU). These metrics include MIoU, which calculates the average IoU across different categories, providing a measure of semantic segmentation accuracy. We also use Mean Squared Error (MSE) to assess the average squared deviation between predicted and actual values, where a lower MSE indicates better accuracy. The SAD measures the absolute differences between corresponding pixels in image blocks, offering an intuitive measure of similarity. For video segmentation, we use the dtSSD and MESSDdt metrics, which account for time-dependent errors and are crucial for evaluating the model’s ability to maintain temporal consistency [31]. Together, these metrics ensure a thorough evaluation of the models’ effectiveness in both image and video segmentation tasks.

(13) $$\mathrm{MIoU}=\frac{1}{k+1}\sum_{i=0}^{k}\frac{p_{ii}}{\sum_{j=0}^{k}p_{ij}+\sum_{j=0}^{k}p_{ji}-p_{ii}}$$

(14) $$\mathrm{MSE}=\frac{1}{m}\sum_{i=1}^{m}{\left(y_{i}-{\bar{y}}_{i}\right)}^{2}$$

(15) $$\mathrm{SAD}=\frac{1}{m}\sum_{t,p}\left|\alpha_{p,t}-{\hat{\alpha }}_{p,t}\right|$$

(16) $$\mathrm{dtSSD}=\frac{1}{m}\sum_{t}\sqrt{\sum_{p}{\left(\frac{d\alpha_{p,t}}{dt}-\frac{d{\hat{\alpha }}_{p,t}}{dt}\right)}^{2}}$$

(17) $$\mathrm{MESSDdt}=\frac{1}{m}\sum_{t,p}\left|{\left(\alpha_{p,t}-{\hat{\alpha }}_{p,t}\right)}^{2}-{\left(\alpha_{p+v_{p},t+1}-{\hat{\alpha }}_{p+v_{p},t+1}\right)}^{2}\right|$$

where i is the true value, j is the predicted value, $p_{ij}$ is the number of predictions from i to j, and k+1 is the total number of categories; $\alpha_{p,t}$ signifies the predicted value at pixel p at time t, and ${\hat{\alpha }}_{p,t}$ represents the true value at pixel p at time t; *m* represents the number of pixels and $v_{p}$ represents the motion vector at pixel p, computed by an optical flow algorithm from the true value time series.

### 3.2. Experimental Results

#### 3.2.1. Ablation Study

In our ablation study, we systematically evaluated the performance improvements of the RVM+ model, focusing specifically on the impact of integrating the ConvGRU module. The goal of this study was to quantify the improvements in segmentation accuracy resulting from this integration. We selected three distinct datasets—VS-test, VMHD_TS, and PhotoMatte85-TS—for comprehensive analysis. These datasets were chosen for their varying levels of complexity and relevance to video portrait segmentation, ensuring a robust evaluation of the model’s capabilities.

Several detailed evaluation metrics were employed to assess the performance of the RVM+ model: MIoU for semantic segmentation accuracy, SAD to measure pixel-level discrepancies, dtSSD to evaluate time-dependent errors crucial for video segmentation, and MSE to quantify prediction accuracy. These metrics provide a multifaceted view of the model’s performance, covering various aspects of segmentation accuracy and error measurement.

The results of this study, presented in Table 2, highlight the performance of the RVM+ model compared to the original version across these metrics. The best-performing metrics for each dataset are highlighted in bold, providing a clear visual representation of the superior performance of the RVM+ model. This comparative analysis demonstrates the improved video portrait segmentation accuracy of the RVM+ model, attributing the improvement primarily to the integration of the ConvGRU module. The study results not only validate the effectiveness of the ConvGRU integration in the RVM+ model but also establish its superiority over the original version in handling complex video segmentation tasks.

#### 3.2.2. Quantitative and Qualitative Results

The results in Table 3 illustrate the performance of our proposed RVM+ model on three video portrait segmentation datasets: VS-test, VMHD_TS, and PhotoMatte85-TS. The model achieves an impressive MIoU of 0.974 on the VS-test set, indicating highly accurate segmentation relative to the ground truth. Its performance drops slightly on the more challenging VMHD_TS and PhotoMatte85-TS datasets, with MIoUs of 0.852 and 0.895, respectively. This decrease may be attributed to the increased complexity or variability within these datasets. The SAD scores are low for all datasets, with the lowest being 5.81 for the VS-test set, suggesting a close match between the segmented portraits and the ground truth. The dtSSD and MSE metrics further corroborate the model’s accuracy, with particularly low MSE scores, the lowest being 0.011 on VMHD_TS, underscoring the model’s consistency in pixel-wise accuracy. Additionally, the bps value indicates the model’s computational efficiency, reaching up to 4.15 M on the VMHD_TS dataset. The FPS value reflects the model’s real-time performance capability, achieving a peak of 32.3 FPS on the VS-test dataset, demonstrating the model’s effectiveness in real-time applications. Taken together, these results indicate that the RVM+ model is highly effective for video portrait segmentation tasks, with some variability in performance across different datasets—typical of model generalization.

In our study, we extended the evaluation of the RVM+ model to include various real-world scenarios. To achieve this, we selected six test videos, each chosen to represent a diverse set of challenges commonly encountered in video segmentation. These challenges included different motion dynamics, lighting conditions, and scene complexity, providing a robust testbed for assessing the model’s capabilities. For each video, we systematically selected three key frames that exemplified these challenges, ensuring a comprehensive and representative assessment of the RVM+ model’s performance across typical and complex situations.

**Figure 10:**
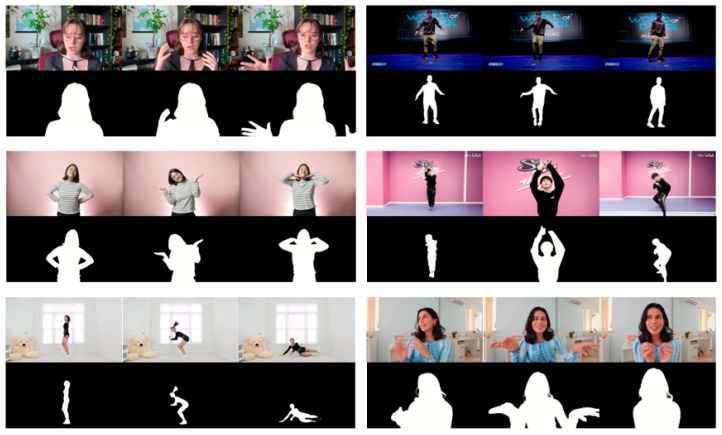
Segmentation results of a three-frame test video.

Enhanced visual representations in Figure 10 provide a side-by-side comparison of the RVM+ model’s segmentation results with the corresponding ground truth data. By juxtaposing the model’s output with the actual data, we offer a clear visual assessment of its performance in different scenarios. This illustrative approach not only highlights the model’s segmentation capabilities but also complements and reinforces the quantitative data presented in our study.

**Figure 11:**
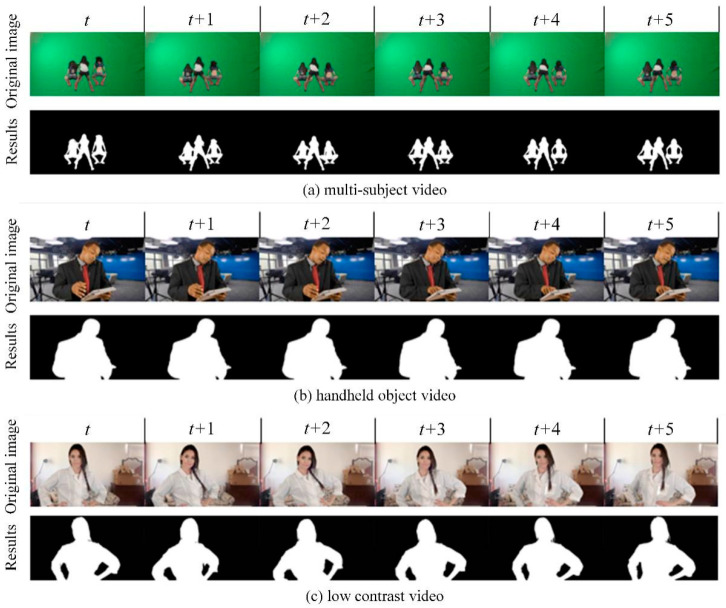
Segmentation results from challenging scenarios.

Furthermore, our study offers a detailed technical analysis of the advanced segmentation model, highlighting its ability to address challenging scenarios commonly encountered in video segmentation. The model employs a sophisticated convolutional neural network architecture augmented with depth-aware layers, which are critical for effectively distinguishing overlapping objects in densely populated scenes—a common issue in multi-subject overlap scenarios. As shown in Figure 11a, this feature enables the model to handle complex multi-subject scenes, ensuring accurate segmentation even when subjects are closely intertwined or overlapping.

In addition to managing static complexity, the model excels in dynamic motion scenarios. It incorporates temporal consistency algorithms that leverage data from adjacent frames, maintaining segmentation accuracy throughout the motion sequence. The integration of optical flow techniques allows the model to dynamically track and adapt to motion, resulting in remarkably accurate segmentation of moving objects, as illustrated in Figure 11b. This capability is essential for videos with moving objects, ensuring consistent and reliable segmentation throughout the sequence.

The model also excels in low-contrast environments, where distinguishing between foreground and background can be particularly challenging. To address this, the model incorporates advanced contrast enhancement techniques, such as adaptive histogram equalization. Additionally, it is rigorously trained on a diverse dataset to ensure robust performance, even when foreground and background colors or brightness levels are closely matched. Figure 11c demonstrates the model’s ability to accurately segment objects in such challenging conditions, showcasing its effectiveness in low-contrast scenarios.

After incorporating advanced features into the segmentation model, we applied knowledge distillation to further optimize its performance. To assess the impact of knowledge distillation, we evaluated the model using the VS-test dataset, with the results presented in Table 4. Our analysis revealed that the accuracy loss following the distillation process was minimal, ranging from 1.0% to 1.5%. This slight decrease in accuracy is considered an acceptable trade-off, particularly given the significant gains in the reduced model size and increased processing speed. These factors are crucial for practical applications where resource efficiency is as important as accuracy.

Additionally, the post-distillation MSE of the model was 0.022, indicating that the model’s ability to predict accurate outcomes was largely maintained despite the reduction in size and complexity. This result highlights the effectiveness of the knowledge distillation process in producing a streamlined yet powerful model, suitable for efficient and accurate video segmentation across various applications.

## 4. Discussion

In our comprehensive evaluation, detailed in Table 5, we compared the performance of five segmentation models—BGMv2 [32], ConnectNet [33], DeepLabV3 [34], MODNet [35], and the extended RVM+ model—across multiple datasets. This comparison was designed to rigorously assess their segmentation accuracy and overall effectiveness in different scenarios. As the results show, the RVM+ model consistently outperformed the other models across all evaluated datasets. One of the notable achievements of the RVM+ model is its remarkable MIoU score of 0.974 on the VS-test set. This high MIoU indicates the model’s exceptional precision in distinguishing objects from the background, a critical aspect of segmentation accuracy. The RVM+ model also maintained the lowest SAD value of 5.81, indicating that the segmented portrait closely matches the ground truth. In contrast, while DeepLabV3 excels in network depth and multi-scale contextual understanding, it falls short in handling fine edges. MODNet achieves fast inference due to its lightweight design but lags behind RVM+ in detail preservation and edge clarity in complex scenes. ConnectNet’s performance falls between these two, showing good accuracy on certain datasets but still trailing behind RVM+ overall. In summary, these results convincingly demonstrate the superior performance of RVM+ in video portrait segmentation tasks, establishing its potential for high-fidelity segmentation applications.

In addition to its MIoU performance, the RVM+ model recorded the lowest SAD score of 5.81, emphasizing its ability to closely match the ground truth and its precision in segmentation. The superiority of the RVM+ model is further confirmed by its performance on other metrics such as dtSSD and MSE. Specifically, on the VMHD_TS dataset, the RVM+ model achieved an MSE of 0.011, reflecting its high accuracy in segmentation tasks. These results underscore the exceptional ability of RVM+ to produce accurate and consistent segmentation, highlighting its adaptability and robustness in addressing diverse segmentation challenges.

**Figure 12:**
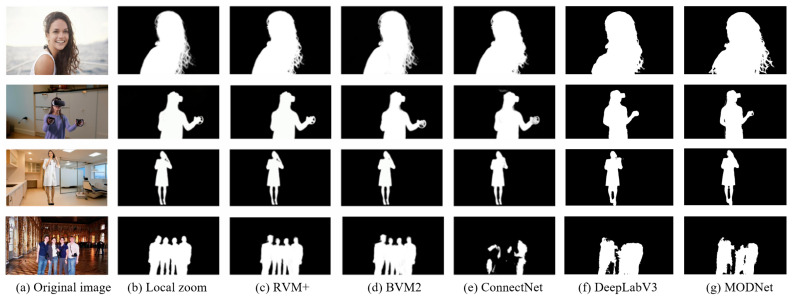
Detailed comparison of segmentation results across different models.

Figure 12 presents a side-by-side visual evaluation of the segmentation quality of three models using frames from validation videos. The original frames provide context for the segmentation challenge, while the local zooms focus on areas that require precise detail handling, such as hair and edges. The RVM+ model’s segmentation is remarkably detailed and accurate, with clean boundaries and well-preserved fine features, outperforming the other models. BGMv2 captures the general shape of the object but shows reduced edge precision, particularly in finer details. ConnectNet’s results exhibit more pronounced inaccuracies, with blurred edges and a loss of detail, indicating lower segmentation accuracy compared to RVM+, which strikes a balance between detail preservation and edge clarity—critical for high-fidelity segmentation. DeepLabV3 is renowned for its deep network architecture and multi-scale context understanding capabilities. It performs well in capturing the large-scale structures of objects but falls slightly short in handling fine edges. MODNet lags behind in detail retention and edge clarity compared to RVM+ in complex scenarios. This contrast highlights RVM+’s superior performance in video portrait segmentation tasks, particularly in balancing detail preservation and edge sharpness, which is essential for high-fidelity segmentation.

**Figure 13:**
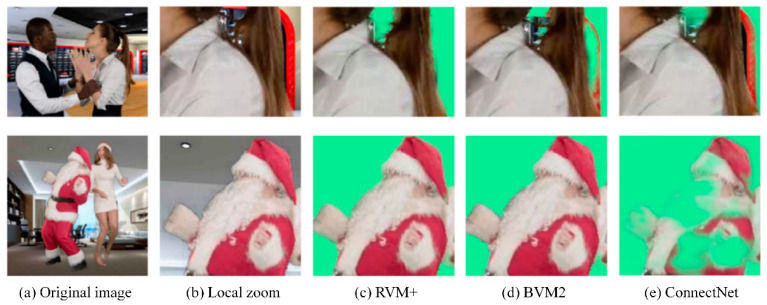
Comparative effects of multi-angle portrait segmentation.

Figure 13 provides a detailed analysis of how RVM+ excels at capturing fine textures, hair strands, and facial details, emphasizing its ability to maintain high-quality segmentation results. BGMv2, while proficient in object segmentation, falls short in contour accuracy, occasionally resulting in minor mis-segmentation or blurred boundaries. ConnectNet, although adequate, tends to be less accurate than both RVM+ and BGMv2, exhibiting noticeable segmentation errors and reduced clarity in subject contours.

Despite these limitations, both ConnectNet and BGMv2 have unique strengths. For example, ConnectNet may offer advantages in certain computing environments, while BGMv2 may perform better in specific segmentation scenarios. Acknowledging these strengths alongside their limitations provides a balanced perspective on the current state of subject segmentation technologies. Comparing these models highlights not only the advances made by RVM+ but also the ongoing evolution of video processing. The results of this study offer valuable insight into potential applications where RVM+ could be particularly beneficial, such as real-time video analysis or complex scene interpretation.

## 5. Conclusions

This study introduces RVM+, an advanced video portrait segmentation model designed to tackle the challenges of dynamic video analysis. By integrating ConvGRUs into the RVM framework, RVM+ enhances temporal consistency and segmentation accuracy, particularly in complex scenarios involving rapid motion, occlusions, and overlapping subjects. Additionally, the implementation of a novel knowledge distillation strategy reduces the model’s computational complexity and size, making it suitable for real-time applications in resource-constrained environments without a significant loss in accuracy. Extensive evaluations on diverse and challenging datasets demonstrate the superiority of RVM+ over state-of-the-art methods. The model consistently delivers high segmentation fidelity, as evidenced by improvements in MIoU, SAD, and temporal-specific metrics such as dtSSD. These results highlight RVM+’s robustness, efficiency, and adaptability to dynamic environments, positioning it as a promising solution for AI-enabled vision sensors. Beyond improved segmentation accuracy, this work contributes to the development of efficient, scalable, and practical solutions for video analysis. Future research will focus on enhancing the model’s generalizability to handle more complex multi-subject and multi-modal scenarios. Moreover, integrating the RVM+ framework with advanced sensing systems, such as IoT devices and wearable sensors, presents exciting opportunities for applications in augmented reality, human–computer interaction, and autonomous systems.

## Figures and Tables

**Table 1:** The Internet’s open-source datasets.

Name	Description
VideoMatte240K	Includes 484 high-resolution alpha masks and foreground video clips, totaling 240,709 frames. The alpha masks and foregrounds were extracted from green screen stock footage from the University of Washington. Of the video clips, 384 are in 4 K resolution, and 100 are in HD resolution.
YouTubeVIS	The large dataset used for video instance segmentation is filtered to include 2985 video clips containing humans.
PhotoMatte85	Includes 85 half-length portraits without backgrounds.
Adobe Image Matting	Provides 214 examples of portrait segmentation.
Supervisely Person Dataset	The dataset consists of 5711 images, including 4079 labeled examples of the human body.
VMD	The dataset includes 39 sets of alpha masks and foreground video clips in three categories: full body, half body, and close-ups of people, totaling 16,698 images.
Distinction-646	We screened 372 images in the dataset that contained people.
BackgroundVideos (BG)	We screened 3279 video sets in the dataset that did not contain characters.

**Table 2:** Ablation study results.

Dataset	Method	MIoU	SAD	dtSSD	MSE
VS-test	RVM	0.959	4.21	2.02	0.037
	RVM+	0.974	5.81	1.78	0.026
VMHD_TS	RVM	0.833	10.36	3.12	0.047
	RVM+	0.852	6.57	1.90	0.011
PhotoMatte85-TS	RVM	0.871	6.52	3.12	0.047
	RVM+	0.895	0.971	1.84	0.014

**Table 3:** Quantitative results on the VS-test, VMHD_TS, and PhotoMatte85-TS datasets.

Dataset	MIoU	SAD	dtSSD	MSE	bps	FPS
VS-test	0.974	5.81	1.78	0.026	3.32 M	32.3
VMHD_TS	0.852	6.57	1.90	0.011	4.15 M	26.7
PhotoMatte85_TS	0.895	0.971	1.84	0.014	3.72 M	28.6

**Table 4:** Comparison of evaluation indices before and after knowledge distillation processing.

Processing	MIoU	SAD	dtSSD	MSE
Before knowledge distillation	0.974	5.81	1.78	0.026
After knowledge distillation	0.962	6.57	1.90	0.022

**Table 5:** Comparison of segmentation results with the different models.

Model	VS-Test				VMHD_TS				PhotoMatte85-TS			
	MIoU	SAD	dtSSD	MSE	MIoU	SAD	dtSSD	MSE	MIoU	SAD	dtSSD	MSE
BGMv2	0.906	25.19	4.51	0.151	0.784	28.50	5.18	0.043	0.761	26.56	4.68	0.051
DeepLabV3	0.884	27.13	6.08	0.213	0.766	30.25	6.23	0.047	0.725	35.27	5.60	0.071
MODNet	0.942	16.34	2.69	0.088	0.846	12.39	3.38	0.039	0.843	10.64	3.25	0.037
ConnectNet	0.955	14.47	2.09	0.035	0.867	17.98	2.74	0.042	0.881	15.88	2.56	0.027
RVM+	0.974	5.81	1.78	0.026	0.852	6.57	1.90	0.011	0.895	0.971	1.84	0.014

## Data Availability

The public VMHD_TS and PhotoMatte85-TS datasets analyzed in this study are available at https://grail.cs.washington.edu/projects/background-matting-v2/#/datasets (accessed on 6 August 2024). The private dataset (VS-test) presented in this article is not readily available, as the raw and processed data required to reproduce these results cannot be shared at this time due to their inclusion in an ongoing study. Requests for access to the dataset should be addressed to LXB at dmia_lab@zcmu.edu.cn.

## References

[1] Wang Y., Zhang W., Wang L., Yang F., Lu H. (2021). Temporal consistent portrait video segmentation. Pattern Recognit..

[2] Garcia-Garcia A., Orts-Escolano S., Oprea S., Villena-Martinez V., Martinez-Gonzalez P., Garcia-Rodriguez J. (2018). A survey on deep learning techniques for image and video semantic segmentation. Appl. Soft Comput..

[3] Seong S., Choi J. (2021). Semantic Segmentation of Urban Buildings Using a High-Resolution Network (HRNet) with Channel and Spatial Attention Gates. Remote Sens..

[4] Du X., Wang X., Li D., Zhu J., Tasci S., Upright C., Walsh S., Davis L. Boundary-sensitive Network for Portrait Segmentation. Proceedings of the 2019 14th IEEE International Conference on Automatic Face & Gesture Recognition.

[5] Hinton G.E., Vinyals O., Dean J.J.A. (2015). Distilling the Knowledge in a Neural Network. arXiv.

[6] Ding H., Jiang X., Shuai B., Liu A.Q., Wang G. Context Contrasted Feature and Gated Multi-Scale Aggregation for Scene Segmentation. Proceedings of the 2018 IEEE/CVF Conference on Computer Vision and Pattern Recognition.

[7] Kim Y.W., Byun Y.C., Krishna A.V.N. (2021). Portrait segmentation using ensemble of heterogeneous deep-learning models. Entropy.

[8] Ma Z., Yao G. (2023). Deep portrait matting via double-grained segmentation. Multimed. Syst..

[9] Yung-Yu C., Curless B., Salesin D.H., Szeliski R. A Bayesian approach to digital matting. Proceedings of the 2001 IEEE Computer Society Conference on Computer Vision and Pattern Recognition.

[10] Shelhamer E., Long J., Darrell T. (2017). Fully Convolutional Networks for Semantic Segmentation. IEEE Trans. Pattern Anal. Mach. Intell..

[11] Ronneberger O., Fischer P., Brox T. U-Net: Convolutional Networks for Biomedical Image Segmentation. Proceedings of the 2015 Medical Image Computing and Computer-Assisted Intervention—MICCAI 2015.

[12] Badrinarayanan V., Kendall A., Cipolla R. (2017). SegNet: A Deep Convolutional Encoder-Decoder Architecture for Image Segmentation. IEEE Trans. Pattern Anal. Mach. Intell..

[13] Zhao H., Shi J., Qi X., Wang X., Jia J. Pyramid Scene Parsing Network. Proceedings of the 2017 IEEE Conference on Computer Vision and Pattern Recognition (CVPR).

[14] Paszke A., Chaurasia A., Kim S., Culurciello E. (2016). ENet: A Deep Neural Network Architecture for Real-Time Semantic Segmentation. arXiv.

[15] Zhao H., Qi X., Shen X., Shi J., Jia J. ICNet for Real-Time Semantic Segmentation on High-Resolution Images. Proceedings of the 2018 European Conference on Computer Vision (ECCV).

[16] Yu C., Gao C., Wang J., Yu G., Shen C., Sang N. (2021). BiSeNet V2: Bilateral Network with Guided Aggregation for Real-Time Semantic Segmentation. Int. J. Comput. Vis..

[17] Howard A., Zhu M., Chen B., Kalenichenko D., Wang W., Weyand T., Andreetto M., Adam H. (2017). MobileNets: Efficient Convolutional Neural Networks for Mobile Vision Applications. arXiv.

[18] Zhou C., Xu C., Cui Z., Zhang T., Yang J. (2022). Self-Teaching Video Object Segmentation. IEEE Trans. Neural Netw. Learn. Syst..

[19] Zheng S., Song Y., Leung T., Goodfellow I. Improving the Robustness of Deep Neural Networks via Stability Training. Proceedings of the 2016 IEEE Conference on Computer Vision and Pattern Recognition (CVPR).

[20] Lin S., Yang L., Saleemi I., Sengupta S. Robust high-resolution video matting with temporal guidance. Proceedings of the 2022 IEEE/CVF Winter Conference on Applications of Computer Vision.

[21] Sun Y., Tang C.K., Tai Y.W. Ultrahigh Resolution Image/Video Matting with Spatio-Temporal Sparsity. Proceedings of the 2023 IEEE/CVF Conference on Computer Vision and Pattern Recognition (CVPR).

[22] Wu J., Jiang Y., Liu Q., Yuan Z., Bai X., Bai S. General object foundation model for images and videos at scale. Proceedings of the 2024 IEEE/CVF Conference on Computer Vision and Pattern Recognition.

[23] Lin S., Ryabtsev A., Sengupta S., Curless B., Seitz S., Kemelmacher-Shlizerman I. Real-Time High-Resolution Background Matting. Proceedings of the 2021 IEEE/CVF Conference on Computer Vision and Pattern Recognition (CVPR).

[24] Dai Y., Price B.L., Zhang H., Shen C. Boosting Robustness of Image Matting with Context Assembling and Strong Data Augmentation. Proceedings of the 2022 IEEE/CVF Conference on Computer Vision and Pattern Recognition.

[25] Zhang X., Yang K., Lu Q., Wu J., Yu L., Lin Y. (2023). Predicting carbon futures prices based on a new hybrid machine learning: Comparative study of carbon prices in different periods. J. Environ..

[26] Hu Y., Lin Y., Wang W., Zhao Y., Wei Y., Shi H. Diffusion for Natural Image Matting. Proceedings of the 2024 European Conference on Computer Vision (ECCV).

[27] Liu H., Soto R.A.R., Xiao F., Lee Y.J. YolactEdge: Real-time Instance Segmentation on the Edge. Proceedings of the 2021 IEEE International Conference on Robotics and Automation (ICRA).

[28] Shorten C., Khoshgoftaar T.M. (2019). A survey on Image Data Augmentation for Deep Learning. J. Big Data.

[29] Siam M., Valipour S., Jagersand M., Ray N. Convolutional gated recurrent networks for video segmentation. Proceedings of the 2017 IEEE International Conference on Image Processing (ICIP).

[30] Gou J., Hu Y., Sun L., Wang Z., Ma H. (2024). Collaborative knowledge distillation via filter knowledge transfer. Expert Syst. Appl..

[31] Ren J., Zhang M., Yu C., Liu Z. Balanced MSE for Imbalanced Visual Regression. Proceedings of the 2022 IEEE/CVF Conference on Computer Vision and Pattern Recognition (CVPR).

[32] Li J., Ohanyan M., Goel V., Navasardyan S., Wei Y., Shi H. VideoMatt: A Simple Baseline for Accessible Real-Time Video Matting. Proceedings of the 2023 IEEE/CVF Conference on Computer Vision and Pattern Recognition Workshops (CVPRW).

[33] Chu L., Liu Y., Wu Z., Tang S., Chen G., Hao Y., Peng J., Yu Z., Chen Z., Lai B. (2021). PP-HumanSeg: Connectivity-Aware Portrait Segmentation with a Large-Scale Teleconferencing Video Dataset. arXiv.

[34] Chen L., Papandreou G., Schroff F., Adam H. (2017). Rethinking atrous convolution for semantic image segmentation. arXiv.

[35] Ke Z., Li K., Zhou Y., Wu Q., Mao X., Yan Q., Lau R.W. (2020). Is a green screen really necessary for real-time portrait matting. arXiv.

